# Placenta Prævia

**Published:** 1880-04

**Authors:** 


### PLACENTA PRÆVIA.

---

Aside from its comparative infrequency, the implantation of the placenta over the mouth of the womb, or placeta prævia—the forecoming of the after-birth—is an occurrence of so serious a nature that it has long claimed the especial attention of the accoucheur. The term "unavoidable hæmorrhage" has been very generally used, since the time of Rigby, as synonymous with placenta prævia; for the reason that hæmorrhage is necessary and almost universal—and, until quite recently, unavoidable and unmanageable. But very rare cases have occurred in which no hæmorrhage was present, not even during the time of labor.

Since the process of ovulation has come to be better understood, the cause of this anomalous implantation is no longer obscure or doubtful, though many theories have prevailed to account for its occurrence. It is generally admitted by the profession that hæmorrhage in these cases, as in all other hæmorrhages caused by the separation of the placenta, is from the uterine sinuses. Prof. J. Y. Simpson, however, held the view that it came from the separated portion of the placenta; but the fact that it usually ceases with the complete separation and expulsion of the placenta, whether this be followed immediately by the birth of the child or not, led him to a correct rule of practice, that to correct the hæmorrhage the placenta should be removed before the child.

The following case of this very formidable accident came under my care on the 11th of February, when I was summoned in haste to see the patient, who had been suddenly taken with profuse hæmorrhage; and the husband expressed his fears that she was about to miscarry, as she had done on a previous occasiou. The period of gestation was between seven and eight months, during nearly the whole of whieh time the woman, aged 35 years, had been in bad health. She had been confined three times previously; and, in her last confinement, as in this, she gave birth to twins. Examination showed the mouth of the womb sufficiently dilated to admit the fingers; and the placenta was found in a central position over the os uteri. I at ence passed the fingers around and separated the placenta throughout nearly its entire extent, and followed this with the administration of ergot. The hæmorrhage soon ceased, or nearly so, and the placenta was expelled. After waiting a little time for further dilatation the membranes were ruptured. To rupture the membranes before dilatation is secured I would regard as bad practice, for we then lose the hydrostatic pressure of the amniotic fluid.

An hour or two after the separation of the placenta the child was born; and, as would be suspected after having so long been deprived of all oxygenation of the blood, it was still-born. Half an hour later the pains were renewed and in a short time another child was born enveloped in in its membranes; these being ruptured, a lusty cry was set up by the infant; when the cord was tied and cut. The placenta belonging to this child was attached to the fundus of the uterus; and, after waiting a little time, was removed by the hand.

The above case is here reported as illustrating the good results which may be expected to follow an artificial separation of the placenta, as soon as the os uteri is dilated sufficiently to admit of this being done. Increased hæmorrhage does not result, for the reason that the uterus is in most cases excited to strong contractions; and the head of the child is forced down upon the open vessels and arrests bleeding by direct pressure immediately upon the detached placenta. The objection to this practice is that the fœtus is deprived of all means of oxygenation of the blood.

The physician is seldom called in till hemorrhage has made its appearance. A vaginal examination is then necessary without delay, to determine the diagnosis—which need be neither difficult nor uncertain—to the end that everything possible may be done looking towards the successful management of the case.

The full period of gestation is seldom completed, hemorrhage generally appearing about the seventh or eighth month; sometimes, though not often, as early as the fifth or sixth month. The nearer the woman approaches to her full time, the greater and more uncontrollable is the hemorrhage; and when it does appear it may be moderate in quantity and soon cease spontaneously for a time, or the discharge may be so great as to place the life of the patient in imminent peril in a few moments.

After hemorrhage has made its appearance, whatever may be the period of gestation, no reliance can be placed on precautionary or conservative treatment. It is then imperatively necessary to empty the uterus, a fact which was recognized by the old practioners, who established what was known as the iron rule, which was given by

Denman as follows: "It is a practice established by high and multiplied authority, and sanctioned by success, to deliver women by art in all cases of dangerous hemorrhage, without confiding in the resources of the constitution."

In a very able and exhaustive paper read before the St. Louis Obstetrical and Genæcological Society (September 19th, 1878), Dr. G. M. B. Maughs says that "In all cases, then, of hemorrhage, when the cause is ascertained to be placenta prævia, whether in the sixth or ninth month, or at term, the uterus must be emptied, as no reliance can be placed in position and hard mattress, cold, acidulated drinks, opium, sugar of lead, cold applied to the vulva, plugging the vagina, etc., further than they may be used as immediate expedients, looking to the speedy delivery of the woman, or promotion of this end; and the physician who would trust his patient to these, with the view of prolonging gestation is but trifling in the presence of fearful danger." In his paper, from which I have drawn freely, Dr. Maughs very ably and fully discusses this whole subject; and the reader cannot fail to be impressed with the views of its author. In reporting the foregoing case it is not my purpose to consider the manner in which the uterus should be relieved of its contents under the various conditions which may present themselves in placenta prævia, though this is of the utmost importance to the well-being of the patient.

